# High-Performance Proteomics Using Nano‑, Capillary‑, and Microflow Chromatographic Separations

**DOI:** 10.1021/acs.jproteome.5c00327

**Published:** 2025-09-03

**Authors:** Giorgi Tsiklauri, Runsheng Zheng, Nicole Kabella, Polina Prokofeva, Christopher Pynn, Bernhard Kuster

**Affiliations:** † School of Life Sciences, 9184 Technical University of Munich, Emil Erlenmeyer Forum 5, Freising 85354, Germany; ‡ 10289 Thermo Fisher Scientific, Germering 82110, Germany

**Keywords:** capillary-flow chromatography, mass spectrometry, kinobeads, phosphoproteomics

## Abstract

Current applications of mass-spectrometry-based proteomics range from single-cell to body fluid analysis, each presenting very different demands regarding sensitivity or sample throughput. Additionally, the vast molecular complexity of proteomes and the massive dynamic range of protein concentrations in these biological systems require highly performant chromatographic separations in tandem with the high speed and sensitivity afforded by modern mass spectrometers. In this study, we focused on the chromatographic aspect and, more specifically, systematically evaluated proteome analysis performance across a wide range of chromatographic flow rates (0.3–50 μL/min) and associated column diameters using a Vanquish Neo HPLC coupled online to a Q Exactive HF-X mass spectrometer. Serial dilutions of HeLa cell line digests were used for benchmarking, and the total analysis time from injection to injection was intentionally fixed at 60 min (24 samples per day). The three key messages of the study are that (i) all chromatographic flow rates are suitable for high-quality proteome analysis, (ii) capLC (1.5 μL/min) is a very robust, sensitive, and quantitative alternative to nLC for many applications, and (iii) showcased proteome, phosphoproteome, and drug proteome data provide sound empirical guidance for laboratories in selecting appropriate chromatographic flow rates and column diameters for their specific applications.

## Graphical abstract

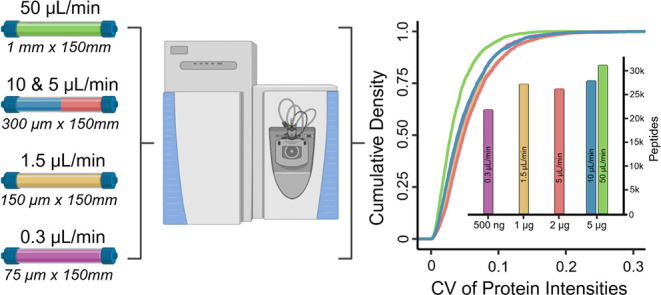

## Introduction

Nanoflow (<1 μL/min) LC-MS/MS (nLC) has been the cornerstone of proteome analysis technology for nearly 30 years due to its exceptional sensitivity when coupled with electrospray ionization mass spectrometry. Over the past decade, significant advancements in mass spectrometric instrumentation have greatly enhanced both the sensitivity and speed of proteome analysis. Progress in sensitivity has led to impressive results when exploring biological systems with only very small quantities of protein available, exemplified by studies utilizing ultralow flow (10–20 nL/min) LC-MS/MS.^1–3^ Improvements in speed have increased the opportunity for applying proteome analysis in a screening mode, such as the analysis of clinical patient cohorts, protein–protein interactions, or protein-drug interactions. In many of these scenarios, sample throughput, robustness, and reproducibility of LC-MS/MS systems are just as important or even more important than sensitivity. These requirements have sparked renewed interest in liquid chromatography separation at higher flow rates^4–6^ because all parameters except sensitivity often see marked improvements when higher flow rate systems are employed. Such systems are often more suitable for high-throughput studies, where exceptional sensitivity may not be of paramount importance. Higher flow alternatives to nanoflow separations have been discussed in detail in a recent review.^7^ Briefly, an elegant study published in 2018 described a microflow LC-MS/MS (μLC) setup (1 mm internal diameter (i.d.) × 25 cm column, 68 μL/min) that identified approximately 2,800 proteins in 1 h from 2 μg of HeLa tryptic peptides using a Q-Exactive Orbitrap mass spectrometer, demonstrating the feasibility of μLC-MS/MS with reasonable sample requirements.^8^ Bian et al.^9^ slightly modified the approach and showed that the proteomic depth of nLC-MS/MS could be matched by microflow LC-MS/MS (μLC-MS/MS) when using 5–10 times more peptide amounts. The same authors^10^ later demonstrated that such a system is extremely robust (>38,000 samples in two years, >14,000 proteomic analyses on a single column). Consequently, μLC-MS/MS has been used in a diverse range of proteomic studies, highlighting its versatility and reliability.^11,12^ Proteomic experiments conducted at even higher (analytical) flow rates have also been reported. Jadeja et al.^13^ recently published proteomic data using 1.5 mm i.d. columns operating at a flow rate of 115 μL/min. Messner et al.^5^ utilized a 2.1 mm i.d. column operating at 800 μL/min using gradients of 30 s to identify new plasma biomarkers of COVID-19 severity. This enabled ultrafast proteomic measurements (several hundred samples per day) of large patient cohorts.

Capillary flow LC-MS/MS (capLC-MS/MS) may also represent an alternative to nLC-MS/MS, but its utility has not been as well explored. Early work by Tao et al.^14^ demonstrated its potential by identifying 1,692 proteins from rat brain tissue using a 300 μm i.d. column operated at 5 μL/min. In 2018, the Ralser laboratory^15^ optimized a 300 μm i.d. column running at 3–10 μL/min and used SWATH-MS to quantify 4,000 human and 1,750 yeast proteins in under 1 h. Similarly, Bruderer et al.^16^ explored a 300 μm i.d. column at 5 μL/min with DIA and managed to analyze 31 human plasma proteomes in 24 h and a total of 1,508 human plasma samples from a nutritional intervention study cohort. Very recently, the ProCan team^17^ in Australia showcased a 300 μm i.d. column setup running at 5 μL/min for high-throughput proteomics and phosphoproteomics of rat tissues using a ZenoTOF instrument, reaching 2,600 protein identifications in a 30 min gradient time from 400 ng rat brain peptides. Also recently, Sui et al.^18^ reported results from comparing μLC-MS/MS (1 mm i.d., 50 μL/min) to capLC-MS/MS (150 μm i.d., 1 μL/min) and concluded that capLC-MS/MS was suitable for high-throughput analysis of clinical samples with limited available material.

While there are several comparative studies showing that reducing the flow rate enhances sensitivity and higher flow rates offer higher throughput and robustness, no single study has compared the performance of nLC-, capLC-, and μLC-MS/MS side by side on the same analytical system. In the past, such a study would have been challenging because chromatographic hardware was typically developed for specific flow rates. In turn, this would have necessitated complex hardware modifications to achieve fair comparisons on a single analytical system. The recent commercialization of the Vanquish Neo LC system now allows such investigations because it can operate at flow rates ranging from 1 nL/min to 100 μL/min without any hardware changes. Therefore, the main purpose of this study was to perform such a comparison and collect empirical data to provide guidance to the scientific community in selecting the most suitable flow rate systems for their specific applications.

## Materials and Methods

### Sample Preparation

Human epithelial cervix carcinoma HeLa cells (ATCC, CCL-2) were cultured in Dulbecco’s Modified Eagle Medium (DMEM; Gibco, Invitrogen), supplemented with 10% fetal bovine serum, 100 U/mL penicillin (Invitrogen), and 100 μg/mL streptomycin (Invitrogen). Cultures were incubated at 37 °C in a humidified atmosphere containing 5% CO_2_. Cells were harvested at approximately 80% confluence by washing twice with PBS and directly lysed on the culture plate using a buffer containing 8 M urea, 80 mM Tris-HCl (pH 7.6), 1 × EDTA-free protease inhibitors (Complete Mini, Roche), and 1 × phosphatase inhibitors (Sigma-Aldrich). The plate was incubated on ice for 5 min before collecting the lysate by scraping. The lysate was centrifuged at 20,000 × g at 4 °C for 30 min, and the supernatant was stored at −80 °C for further analysis. HeLa proteins were digested according to the SP3 protocol.^19^ The obtained peptides were desalted by using the HLB desalting cartridge. Peptides were quantified using the Pierce Quantitative Fluorometric Peptide Assay, dried using SpeedVac, and stored at −20 °C.

Human body fluid specimens for this study were collected with informed consent and in compliance with the ethics approval process of the Medical Faculty of LMU Munich, which granted a waiver for the procedures involving human materials (Reg. No. 23-0491 KB). Blood plasma (1 mL) was collected from a healthy donor and centrifuged at 4,000 g for 10 minutes at 4 °C to obtain the supernatant. A 50 μL aliquot of the supernatant was diluted 5-fold with 8 M urea containing 80 mM Tris-HCl (pH 7.6). The sample was further diluted with five volumes of 40 mM Tris-HCl buffer. For reduction, 1 M dithiothreitol (DTT) was added to achieve a final concentration of 10 mM, and the mixture was incubated at 37 °C for 60 minutes. Alkylation was performed by adding chloroacetamide (CAA) to a final concentration of 55 mM, followed by incubation at room temperature in the dark for 30 minutes. Proteins were digested using a two-step trypsin digestion protocol (4 h in the first step and then overnight) with a protease-to-protein ratio of 1:100 (w/w) for each step. Desalting was carried out using the C18 StageTip protocol, packing one C18 disc (CDS) of ∼1.2 mm diameter into a 200 μL volume pipet tip. Cerebrospinal fluid (CSF) samples were obtained from 10 individuals without neurological abnormalities, and samples were pooled and stored at −80 °C until further use. Proteins were digested by following the SP3 protocol described above for HeLa cells. Desalting was carried out using the C18 StageTip protocol. Peptides were quantified using the Pierce Quantitative Fluorometric Peptide Assay.

### Phosphopeptide Enrichment

In total, 500 μg of HeLa protein digest was separated on a 2.1 × 150 mm Waters XBridge BEH130 C18 3.5 μm column at a flow rate of 200 μL/min. Buffer C was 100% ultrapure water (ELGA), buffer D was 100% acetonitrile (ACN), and buffers A and B were not used in this system. Separation was performed using a linear gradient from 4% D to 32% D over 45 min, ramped to 80% D in 6 min, and held at 80% D for 3 min before being ramped back to 5% D in 2 min. 96 fractions were collected at 0.5 min intervals. Peptides were pooled in a stepwise fashion from 96 to 48 to 24–12 fractions by combining fraction 49 with fraction 1, fraction 50 with fraction 2, and so forth. Fractions were dried in SpeedVac and stored at −80 °C until subsequent phosphopeptide enrichment. Phosphopeptides were enriched from each of the 12 fractions using Fe­(III)-IMAC-NTA (Agilent Technologies) on the AssayMAP Bravo Platform (Agilent Technologies). IMAC cartridges were primed with 100 μL of 99.9% ACN/0.1% TFA and equilibrated with 50 μL of loading buffer (80% ACN/0.1% TFA). Samples were reconstituted in 200 μL of loading buffer, loaded onto cartridges, and washed with 50 μL of loading buffer. Phosphopeptides were eluted with 40 μL of 1% ammonia, quantified using NanoDrop 2000 (Thermo Scientific), dried down, and stored at −80 °C until subjected to LC-MS/MS analysis. 500 ng peptide loading was used per injection in both capLC and nLC-MS/MS systems.

### Kinobeads Pulldowns

Kinobeads selectivity profiling of the multikinase inhibitor AT-9283 was conducted using a standard published protocol.^20^ Briefly, K-562 (ATCC, CCL-243), COLO-205 (ATCC, CCL-222), and MV-4-11 (ATCC, CRL-9591) cells were cultured in RPMI 1640 medium (Biochrom GmbH) supplemented with 10% (v/v) FBS (Biochrom GmbH) and 1% (v/v) antibiotics. SK-N-BE(2) (ATCC, CRL-2271) cells were grown in DMEM/Ham’s F-12 (1:1) supplemented with 10% or 15% (v/v) FBS, respectively, and 1% (v/v) antibiotics (Sigma). OVCAR-8 (RRID: CVCL_1629) cells were cultured in IMDM medium (Biochrom GmbH) supplemented with 10% (v/v) FBS. Cells were lysed in a buffer containing 0.8% NP-40, 50 mM Tris-HCl (pH 7.5), 5% glycerol, 1.5 mM MgCl_2_, 150 mM NaCl, 1 mM Na_3_VO_4_, 25 mM NaF, 1 mM DTT, protease inhibitors (SigmaFast), and phosphatase inhibitors (prepared in-house following Sigma-Aldrich’s cocktail 1, 2, and 3 protocols). A pooled lysate (2.5 mg protein) from all five cell lines was preincubated with AT-9283 at increasing concentrations (DMSO vehicle, 3 nM, 10 nM, 30 nM, 100 nM, 300 nM, 1 μM, 3 μM, 30 μM) for 45 min at 4 °C in an end-over-end shaker. Kinobeads (18 μL settled volume) were added to the lysate-compound mixture and incubated for 30 min at 4 °C with end-over-end agitation. After washing, bead-bound proteins were reduced with 50 mM DTT in 8 M urea, 40 mM Tris-HCl (pH 7.4) for 30 min at room temperature, alkylated with 55 mM CAA, and digested with trypsin. Peptides were desalted using SepPak C18 μElution plates (Waters) and dried in SpeedVac prior to LC-MS/MS analysis. 500 ng of peptide loading was used per injection in both capLC and nLC-MS/MS systems.

### LC-MS/MS Analysis

All LC-MS/MS analyses were performed on a Vanquish Neo LC system coupled to a Q Exactive HF-X mass spectrometer (Thermo Fisher Scientific) operating in positive polarity mode. Reverse-phase chromatography was performed using 150 mm long columns of varying i.d. (75, 150, 300, 1,000 μm) packed with identical material (Acclaim PepMap Neo C18, 2 μm particle size, 100 Å pore size, Thermo Fisher Scientific). Solvent A was 0.1% FA/3% DMSO (v/v) in water; solvent B was 0.1% FA/3% DMSO (v/v) in 100% ACN. DMSO was increased to 5% for flow rates <1.5 μL/min. The column temperature was maintained at 55 °C. Between runs, columns were equilibrated with three column volumes of 1% solvent A. A 25 μL sample loop was used for all direct injection setups, and 10 μL volume was injected. Data-dependent acquisition (DDA) was employed using a full MS-ddMS2 method (S-lens RF level 40, 360–1300 *m*/*z* MS1 scan range, full MS AGC target 3E6, max injection time (IT) of 50 ms; for MS2 scans: 1.3 *m*/*z* isolation width, 100 *m*/*z* fixed first mass, max IT 22 ms, higher-energy collisional dissociation (HCD) fragmentation with a normalized collision energy (NCE) of 28, charge states from +2 to +5, peptide match set to preferred, and isotope exclusion on). MS1 and MS2 spectra were acquired in profile and centroid mode, respectively. To avoid issues with potential sample carryover, samples of the dilution series were measured starting with the lowest sample quantity. Blanks between samples were run at loadings of 20 ng or higher for nLC and capLC, and 500 ng or higher for μLC separations. Experiments for the evaluation of different flow rates were done in decreasing order of flow rate: 50 μL/min first and 0.3 μL/min setup last. The mass spectrometer was cleaned before running the 50 μL/min samples, again before running 5 μL/min, and again before 1.5 μL/min samples. Further setup-specific details are provided below.

μLC – 50 μL/min: Samples were analyzed using an Acclaim PepMap Neo C18 column (1 mm i.d. × 150 mm, P/N 164711) with a 54 min gradient (1–3.3% B in 1 min, 3.3–20% B in 45.1 min, 20–28% B in 6 min, 28–90% B in 0.9 min, and wash at 90% B for 2 min) at 50 μL/min. Sample loading, equilibration, and washing were performed at 100 μL/min. 50 μm i.d. nanoViper capillaries connected the LC system to the Ion Max API source (HESI-II probe, depth set to A line). MS settings: spray voltage 4.0 kV, capillary temperature 320 °C, vaporizer temperature 200 °C, sheath/aux/sweep gas flow rates 32/5/0, full MS resolution 60,000 (at *m*/*z* 200). MS2 settings: resolution 15,000, intensity threshold 9E4, AGC target 1E5, Top12 method, and dynamic exclusion 25 s.

μLC – 10 μL/min: Samples were analyzed using an Acclaim PepMap Neo C18 column (300 μm i.d. × 150 mm, P/N 164537) with a 54 min gradient (same as the 50 μL/min method above) but at a 10 μL/min flow rate. Sample loading, equilibration, and washing were performed at 15 μL/min. NanoViper capillary connections were the same as for the 50 μL/min setup. MS settings were identical to the 50 μL/min setup, except: spray voltage 2.5 kV, capillary temperature 320 °C, vaporizer temperature 72 °C, sheath/aux/sweep gas flow rates 8/2/0, Top18 method, and dynamic exclusion set to 30 s.

capLC – 5 μL/min: Identical to the 10 μL/min setup, except 5 μL/min flow rate during the gradient, a spray voltage of 2.3 kV, a vaporizer temperature of 63 °C, and sheath/aux/sweep gas flow rates of 8/1/0.

capLC – 1.5 μL/min: Samples were analyzed using an Acclaim PepMap Neo C18 column (150 μm i.d. × 150 mm, P/N DNV150150PN) with a 50 min gradient (1–3.3% B in 1.5 min, 3.3–12% B in 26.1 min, 12–20% B in 15.5 min, 20–28% B in 4.5 min, 28–90% B in 0.8 min, and wash at 90% B for 1.6 min) at 1.5 μL/min. Sample loading, equilibration, and washing were performed at 3 μL/min. Twenty μm i.d. nanoViper capillaries connected the pump, valves, and column. The column outlet was directly interfaced with the Nanospray Flex source (30 μm i.d. steel emitter) using a nanoViper-to-open silica capillary tube adapter (P/N 6041.5290). MS settings: spray voltage 3 kV, capillary temperature 275 °C, and full MS resolution 60,000 (at *m*/*z* 200). MS2 settings: resolution 15,000, intensity threshold 9E4, AGC target 1E5, Top18 method, and dynamic exclusion at 40 s. For the measurement of phosphopeptides, MS2 settings: max IT was changed to 50 ms, and for analyzing kinobeads pulldown experiments, the Top12 method was used instead.

nLC – 0.3 μL/min: Samples were analyzed using an Acclaim PepMap Neo C18 DNV column (75 μm i.d. × 150 mm, P/N DNV75150PN) with a 45 min gradient (1–3% B in 3.3 min, 3–10% B in 22 min, 10–20% B in 14.7 min, 20–35% B in 3.3 min, 35–90% B in 0.5 min, and wash at 90% B for 1.2 min) at 0.3 μL/min. Sample loading, equilibration, and washing were performed at 1.0 μL/min. Twenty μm i.d. nanoViper capillaries connected the pump, valves, and column. The column outlet was interfaced with the Nanospray Flex Ion source (30 μm i.d. steel emitter) using a nanoViper-to-open silica capillary tube adapter (P/N 6041.5290). MS settings: spray voltage 2.1 kV, capillary temperature 275 °C, and full MS resolution 120,000 (*m*/*z* 200). MS2 settings: resolution 15,000, intensity threshold 9E4, AGC target 1E5, Top24 method, and dynamic exclusion 40 s. For the measurement of phosphopeptides, MS2 settings: max IT was changed to 50 ms, and for analyzing kinobeads pulldown experiments, the Top12 method was utilized instead.

Multinozzle Setups: For the 5 and 1.5 μL/min capLC setups, an MnESI source (Newomics) was used, employing the M3 8-nozzle 10 μm i.d. emitter. An ESI spray voltage range of 3.5–4.5 kV was tested for both flow rates. For 5 μL/min, 3.7–4.3 kV showed high uniformity in ESI plumes; 4.2 kV was chosen in the end. For 1.5 μL/min, 3.8–4.4 kV showed higher stability, and 4.3 kV was the final choice. The MS was operated as follows: capillary temperature 320 °C; vaporizer temperature 30 °C. The flow rates of sheath gas, auxiliary gas, and sweep gas were set to 1, 0, and 0, respectively. The LC gradient for the 5 μL/min multinozzle setup was reduced to 52 min (3.3–20% B in 44 min, 20–28% B in 6 min, 28–90% B in 2 min) to accommodate slower sample loading, equilibration, and washing speed at 10 μL/min, which was necessary for continuous spray stability. All other LC and MS parameters were identical to the respective flow rate setups above.

Additional notes: LC-MS system maintenance when using DMSO or high solvent flow rates: Some scientists in the field believe that the introduction of DMSO into the mass spectrometer increases the frequency at which instruments need to be cleaned. To clarify, this is not the case. The authors used DMSO in almost all of their LC-MS systems for 13 years and on every Orbitrap generation available within that time. DMSO is a volatile solvent, and as long as the ESI source region and the first part of the vacuum system are maintained at a high enough temperature, the presence of DMSO is not an issue in terms of system cleaning. Similarly, some scientists in the field believe that higher flow LC rates could require more frequent mass spectrometer cleaning. The opposite is the case. Personal experience and that of field service engineers show that nLC actually makes instruments dirty more quickly than systems operating at higher flow rates. This is because most of the volatile solvent is pumped away as neutral gas, while the ionization of nonvolatile peptide samples is much more efficient using nLC than higher flow rates. In our experience, two other factors are more important in this context: first, the type of sample measured (e.g., phosphopeptides and FFPE samples are considered “dirtier” than, e.g., full proteome digests of cell lines). Second, the type of instrument used. For instance, (for unknown reasons), Orbitrap Eclipse instruments require less cleaning than Exploris 480 systems, even though their front-end parts are essentially the same.

Additional notes: LC-MS acquisition parameters: For consistency, we only compared LC-MS setups with direct sample injection. Due to the longer loading/equilibration times associated with direct sample injection, the gradient time of the nLC setup was 10% shorter than that of, for example, the capLC setup. In addition, we used the same 30 μm i.d. electrospray emitter for the nLC setup and a 1.5 μL/min capLC setup. For the former, a smaller i.d. emitter would offer advantages in terms of postcolumn dead volume, and using columns with integrated emitters would largely eliminate this effect. However, these were excluded from this study because of the following: a) integrated emitter columns were not available for the column material and dimensions used here. As a result of these compromises, the overall performance of the nLC setup was likely not as high as it could have been under optimal conditions. Another consideration is that the instrument control software of the HF-X mass spectrometer does not support setting a fixed cycle time for switching between MS1 and MS2 spectra. Therefore, the topN approach had to be used. Our initial work on the μLC setup (50 μL/min) provided a detailed empirical optimization of the topN approach.^9^ We re-examined this parameter for the current study and found Top12 to still be optimal. For the 10, 5, and 1.5 μL/min setups, LC peaks were wider, which allowed for increasing the number of MS2 scans to Top18. We also tested Top30, but this did not provide any further benefit. For the nLC setup, we used the standard Top24 method that had been independently established and is a standard in the laboratory of the authors. We also note that the nLC setup used an MS1 resolution of 120,000, whereas all other methods employed a resolution of 60,000. This difference would conceptually put the nLC setup at a disadvantage, but the actual impact on the results was negligible (Figure S1). The longer scan time of the Orbitrap at 120 k resolution essentially reduced the number of MS2 scans per cycle (14%). However, the nLC setup collected substantially more MS2 scans at loadings of 5–50 ng and only 7% fewer at 100 ng. At all of these loadings, more peptide and protein identifications were obtained by the 1.5 μL/min capLC setup. This implies higher overall quality of MS2 spectra in the capLC setup even at low sample loadings, which is presumably the result of the sharper LC peaks, leading to higher sample concentration, which is beneficial for electrospray ionization efficiency. We, therefore, conclude that the higher MS1 resolution setting did not have a detrimental impact on the performance of the nLC setup. Loss of MS2 scans was more pronounced at loadings of 200 and 500 ng (18%) which may be due to the combination of higher MS1 resolution and shorter gradient time.

### Raw MS Data Processing and Analysis

Raw data files were processed using MaxQuant^21^ (v1.6.2.10) and searched against a human FASTA database (UniProtKB release 07.2019, UP000005640_74349) using default settings. Briefly, trypsin was set as the enzyme with up to two missed cleavages. Cysteine carbamidomethylation was configured as a fixed modification, while N-terminal acetylation and methionine oxidation were specified as variable modifications. The false discovery rate (FDR) was maintained at 1% at the site, peptide-spectrum match (PSM), and protein levels. Chromatographic full width at half-maximum (FWHM information was extracted from the MaxQuant output file “allPeptides.txt”. Andromeda scores were taken from the “evidence.txt” file after excluding all reverse sequences and potential contaminants. Peptide intensity boost comparisons were done on data from the MaxQuant output file “peptides.txt” using the Benjamini-Hochberg multiple testing correction procedure^22^ set to 0.05 FDR. The analysis included only peptides that were present (had intensity values) in all three replicates of the reference condition (50 μL/min) and were also consistently detected across all three replicates of the tested condition. The peak capacity was calculated according to the following formula:^8^

$$Pc\left(peak\;capacity\right)=1+\frac{t_{g}\left(\mathrm{gradient}\;time\right)}{1.679xW50\%}$$

where *W*50% is the median width of the chromatographic peak for all peptides.

For analyzing kinobeads pulldown data, the CurveCurator pipeline^23^ was employed, permitting up to six missing values, using a fold change minimum of 0.5 and an alpha value cutoff of 0.01. pEC_50_ values (= −log_10_EC_50_) were obtained from CurveCurator analysis, and apparent dissociation constants (*K*_d_^app^; often expressed as p*K*_d_^app^ = −log_10_ *K*_d_^app^) were subsequently calculated by multiplying EC_50_ values by correction coefficients, defined as the ratio of pulldown experiment intensity values to DMSO control. Percent carryover in the robustness tests was calculated by dividing the total protein intensity sum in the blank by the same metric from the previous LC-MS run. Data analysis and visualization were performed using in-house developed R and Python scripts, along with Skyline^24^ (v22.2), BioRender, and Inkscape.

## Results and Discussion

### Study Design for the Side-by-Side Performance Comparison of Proteome Analysis by μLC-, capLC-, and nLC-MS/MS

The overall design of this comparison is summarized in Figure 1A. The key principles driving this design were to perform all experiments using (i) the same samples, (ii) the same HPLC, (iii) the same column material and length, (iv) the same mass spectrometer, (v) the same sample throughput, and (vi) the same analysis software.

**Figure 1:**
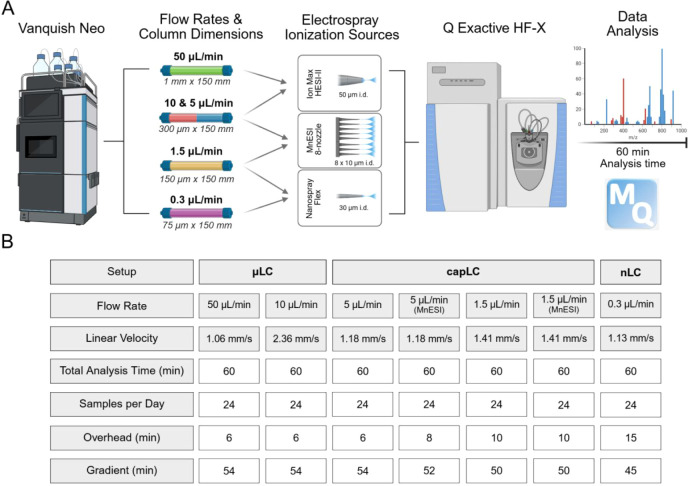
(A) Study design for the comparative performance evaluation of μLC-, capLC-, and nLC-MS/MS systems for proteome analysis. (B) Tabular summary of key LC parameters for each setup.

The Vanquish Neo UHPLC system accommodated all five flow rates tested in this study, and all separations were performed using commercially available Acclaim PepMap Neo C18 columns as follows: μLC (50 μL/min: 1 mm i.d. × 150 mm), capLC (10 μL/min: 300 μm i.d. × 150 mm; 5 μL/min: 300 μm i.d. × 150 mm; 1.5 μL/min: 150 μm i.d. × 150 mm), and nLC (0.3 μL/min: 75 μm i.d. × 150 mm). All analytical columns had the same length, featured identical particle size and chemistry (2 μm/100 Å, C18), and only differed by internal diameter. Depending on the setup, coupling of the Vanquish Neo with the Q Exactive HF-X mass spectrometer was achieved by using either the Ion Max API source with a HESI-II probe (for 50, 10, and 5 μL/min flow rates) or the Nanospray Flex ion source (for 1.5 and 0.3 μL/min flow rates). An add-on to the study was the use of a commercial multinozzle electrospray emitter for the 1.5 and 5 μL/min flow rates.

To assess the sensitivity of each setup, serial dilutions of a HeLa cell line digest (5 ng–10 μg on column) were analyzed in triplicate. Instead of fixing the analytical gradient to a uniform length, all comparisons were made by using the increasingly popular “samples per day” (SPD) approach. This concept proved to be practical, particularly when planning larger-scale projects. To allow for comprehensive sampling, the injection-to-injection time was fixed at 60 min (24 SPD) for each flow rate throughout this study. The LC systems were operated in direct sample injection mode. A consequence of using the SPD approach was that the analytical gradient times and overhead times (sample loading and column equilibration) were not the same for all setups (Figure 1B). This was due to the longer sample loading (10 μL volume) and equilibration times required for the direct injection gradient flow rates of 1.5 and 0.3 μL/min. While this is suboptimal for low flow rates (particularly for a flow rate of 0.3 μL/min) in terms of overhead times, we maintained this setup for (i) consistency, (ii) minimizing sample evaporation in 96-well sample plates, and (iii) ensuring the same sample volume is injected every time. LC parameters were individually optimized for each flow rate, and gradient shapes were tailored to make separations as uniform as possible (Figure S2). MS parameters were adjusted to match LC characteristics (see Methods for details).

### Comparison of the Sensitivity of Proteome Analysis by μLC-, capLC-, and nLC-MS/MS

The sensitivity of each setup was assessed by analyzing serial dilutions of HeLa cell line digests in triplicate, initially using the number of identified peptides and proteins as a metric (Figure 2A,B). The observed general trends align well with expectations, such that lower flow rates (≤1.5 μL/min) outperform higher flow rates for peptide loadings below 1 μg, where electrospray ionization (ESI) efficiency drives sensitivity. Conversely, higher flow rate separations require a higher sample loading to compensate for the loss of ESI efficiency. The observed increase in the identified peptides and proteins with increasing loading amounts allowed us to determine an optimal range of peptide loading for each flow rate setup. The observed saturation serves as a valuable indicator for estimating the upper limit of the appropriate sample amount for each system. Identifying the optimum range is of practical value, as injecting too little results in lower IDs, while injecting too much can exceed column capacity, leading to deterioration of chromatographic peak shapes, fewer IDs, and poorer quantification.^26^ Based on the empirical data, 10–50 μL/min flow rates are best for sample loadings of ≥5 μg, and 5 μL/min is effective for 1–5 μg. Interestingly, 1.5 and 0.3 μL/min showed very similar performance in the 10 to 500 ng range, with consistently higher numbers for the 1.5 μL/min capLC system starting from 100 ng loading. As a second metric for comparing the sensitivity of the different systems, we used the chromatographically integrated peptide precursor ion intensity measured by the mass spectrometer (area under the curve, AUC of the extracted ion chromatogram, XIC). To ensure fair comparison, we fixed sample loading to 200 ng, ensuring that all systems were below the saturation point. The 50 μL/min setup was used as a reference, and peptides detected in this setup (in all three replicates) were used to calculate the intensity differences of the same peptides between the different flow rates (FDR = 0.05, Benjamini-Hochberg procedure). As shown in Figure 2C, reducing the flow rate from 50 to 10 μL/min resulted in a median 70% increase in peptide intensity. Further reduction to 5 μL/min yielded a 270% boost. A major change occurred at a flow rate of 1.5 μL/min, which achieved a 1,300% increase in intensity, and a further improvement to 2,210% was reached using nLC operated at 0.3 μL/min. At higher sample loadings, the intensity boosts observed for lower flow rates (≤1.5 μL/min) strongly diminished. This is likely due to either column overloading, ESI saturation, or both (Figure S3).

**Figure 2:**
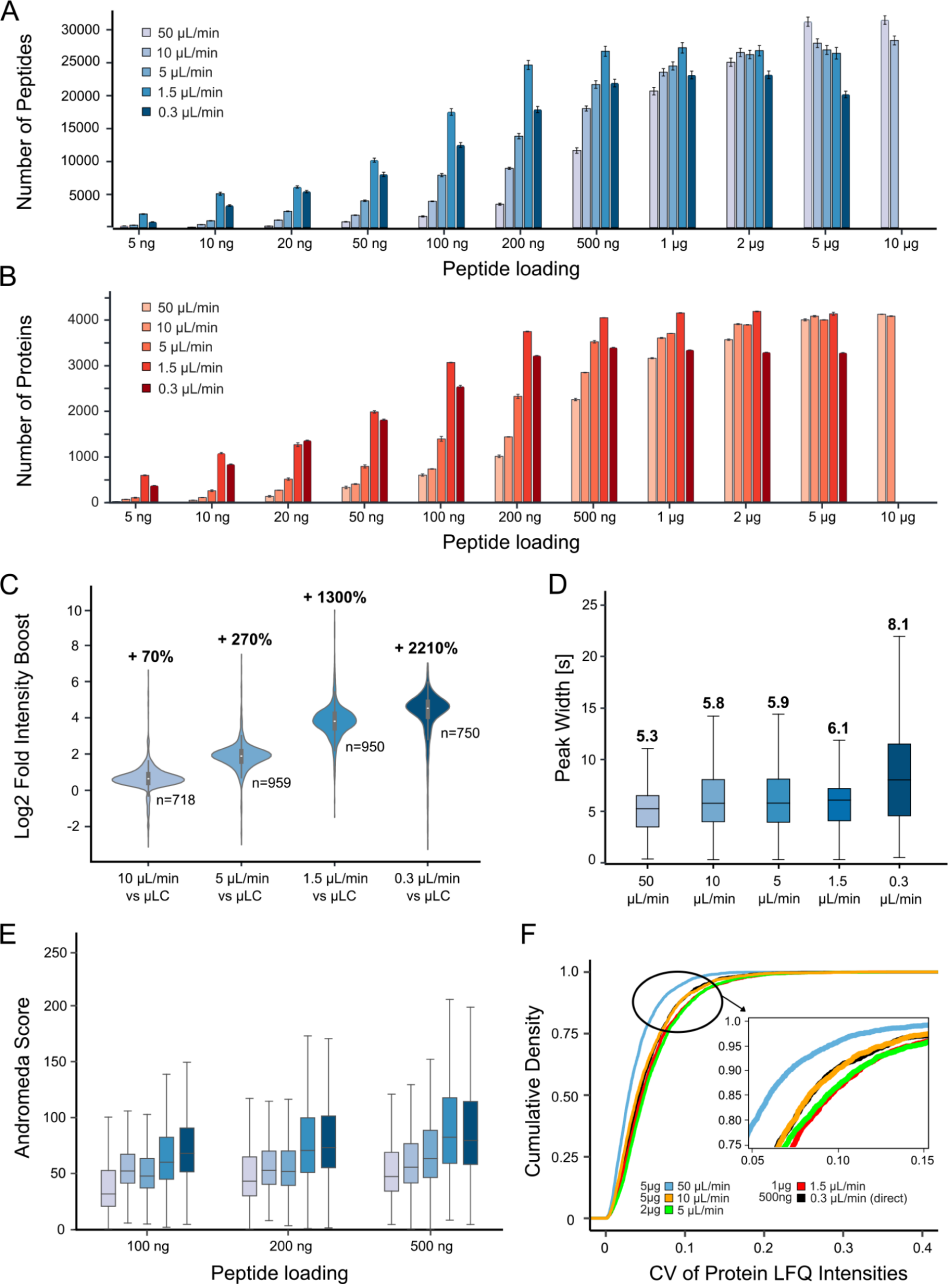
Comparison of all LC-MS/MS setups based on serial dilution experiments (*n* = 3 measurements for each sample loading). (A) Bar plot showing the average number of identified peptides for each sample loading. (B) Same as (A) but for protein groups (red). (C) Violin plots showing the distribution of the relative boost of peptide intensities (based on the area under the curve (AUC) of the extracted ion chromatograms) for all setups at 200 ng of peptide loading compared to the reference μLC setup operating at a flow rate of 50 μL/min. Numbers above violin plots represent % change relative to the 50 μL/min reference; *n* = total number of overlapping peptides that were identified in all replicates of the reference and the flow-rate system tested; color code as in (A). (D) Box plots showing the distributions, medians, and interquartile ranges of chromatographic peak widths at half-maximal signal (FWHM) of all identified peptides at optimal column loadings; color code as in (A). (E) Box plots showing the distributions, medians, and interquartile ranges of MaxQuant Andromeda scores of all setups at three levels of peptide loading; color code as in (A). (F) Cumulative density plot illustrating the quantitative repeatability (precision) of all setups measured by the coefficient of variation (CV) of quantified proteins at optimal peptide loadings.

The observation that the 1.5 μL/min capLC setup was on par with or even outperformed the 0.3 μL/min nLC setup in terms of peptide and protein identifications was surprising, given that the nLC system exhibited higher sensitivity (∼70% higher median peptide intensity). However, the analytical gradient time of the nLC setup was 5 min shorter (10%) due to the longer overhead time required for sample loading and system equilibration. Still, capLC prevailed because of its superior chromatography, evidenced by substantially sharper peaks (median FWHM of 6.1 vs 8.1 s; a difference of 33%) and narrower peak width distribution (Figure 2D) leading to higher estimated median peak capacities (294 vs 200, a difference of 47%; Figure S4). The reason for the overall better separation by capLC should not be attributed to the nLC column being overloaded, as chromatographic peak widths did not vary substantially for the different loadings (except when saturating the respective column). Given that the same column material was used for all experiments, mass transfer should not contribute to the observed differences in separation performance. We also calculated the linear flow velocities for all setups (Figure 1B) and found them to be very similar. Since nLC columns can be more difficult to pack uniformly, there may be more longitudinal diffusion in nLC columns compared to capLC columns. Another, and perhaps stronger, factor is postcolumn mixing caused by postcolumn dead volumes, which are more detrimental for nLC than capLC separations. All these factors together resulted in a higher peak capacity for capLC vs nLC (Figure S4).

The improved separation also comes with the effect of a higher peptide concentration, which improves ESI response, particularly for low-abundance peptides, in turn leading to higher signal intensities. This increases the probability of triggering a data-dependent scan and doing so closer to the apex of the LC peak.^26^ Higher peptide signals also result in more precursor ions for fragmentation within a given time frame, leading to enhanced quality of MS2 spectra and, ultimately, more peptide and protein identifications. This interpretation is supported by the very similar Andromeda score distributions of identified peptides in the 1.5 μL/min capLC and 0.3 μL/min nLC systems, starting from 200 ng loading (Figures 2E and S3).

Reproducible peptide and protein identifications, as well as robust quantification in proteomic experiments, are at least as important as, if not more important than, covering a large number of proteins. When examining replicate measurements of optimal peptide loading examples, all setups showed very good and similar identification reproducibility (Figure S5). More specifically, 58%, 54%, 53%, 47%, and 51% of all peptides were found in all replicates for the 50, 10, 5, 1.5, and 0.3 μL/min flow rates, respectively. The corresponding figures for protein groups are 87%, 85%, 84%, 83%, and 82%.

The 50 μL/min system showed by far the best overall quantitative precision, with a coefficient of variation (CV) of <10% for >95% of all quantified proteins. Nevertheless, when peptide loading was adjusted to an appropriate level, all evaluated chromatographic setups achieved good quantitative precision (<20% CV for >95% of all quantified proteins; Figure 2F).

### Performance Evaluation of a Multinozzle ESI Source

To evaluate whether the performance of the capLC systems (1.5 and 5 μL/min) could be further improved, we tested a commercial multinozzle ESI source (MnESI, Newomics), which splits the LC flow into eight streams, effectively reducing the flow delivered to each emitter nozzle by 8-fold (M3 Emitter). The underlying concept is to enhance ESI efficiency by reducing the flow rate postcolumn while maintaining full chromatographic performance. Kreimer et al.^27^ recently reported results using such a multinozzle emitter in conjunction with a 300 μm i.d. × 50 mm C8 column running at 9.5 μL/min and a dual-trap setup to analyze plasma and cell digest samples by data-independent acquisition (DIA) on a timsTOF mass spectrometer at a rate of 15 min/sample. They identified 400 proteins in plasma and 4,000 proteins in cell digests. However, no comparison to a reference system without a multinozzle emitter was provided.

Here, we again used serial dilutions of HeLa cell digests to compare the capLC setups with and without a multinozzle emitter side by side. The 5 μL/min setup showed a benefit in the number of peptide (up to 16%) and protein (up to 18%) identifications (Figure 3A,B), even though the available gradient time was two min shorter than on the reference system. For the 1.5 μL/min setup, the MnESI source performed substantially worse in terms of peptide and protein identifications than the reference NanoFlex source (Figure 3C,D), which was also reflected by lower median peptide intensities (Figure 3E). Chromatographic performance of the MnESI and reference systems was nearly identical as expected (Figure 3F), and no strong differences were detected when comparing quantitative precision (Figure 3G). Therefore, the observed differences can be attributed to the ionization source itself. At a flow rate of 5 μL/min, the MnESI reduces flow to 0.625 μL/min per nozzle, possibly enhancing ESI efficiency. An additional or alternative contributing factor is that the MnESI’s emitter nozzles have a 10 μm i.d. tip opening, while HESI needles has a 50 μm i.d. tip opening. The smaller emitter i.d. should work in tandem with the reduced flow rate to promote smaller droplet formation, thereby improving ESI efficiency. In contrast, for the 1.5 μL/min setup, transitioning from a NanoFlex 30 μm i.d. steel emitter to 10 μm i.d. multinozzle emitters proved ineffective. At a flow rate of 1.5 μL/min, the MnESI reduces the flow per nozzle to 0.188 μL/min. It is possible that the 8-nozzle 10 μm i.d. emitter architecture is no longer efficient for a flow rate this low. It can be anticipated that the recently released 5-nozzle 10 μm i.d. MnESI emitter should perform better in the 1.5 μL/min flow rate regime.

**Figure 3:**
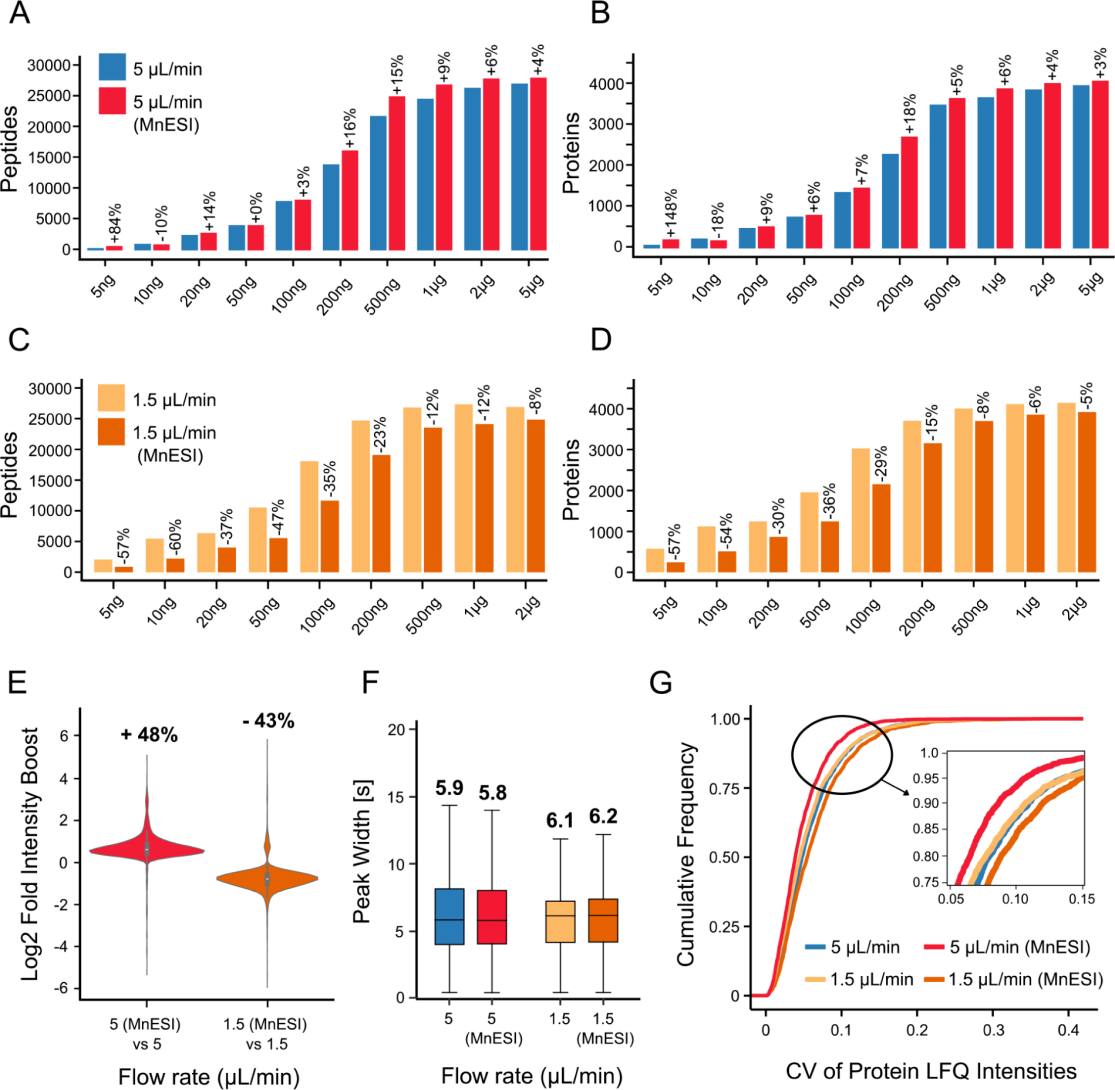
Benchmarking results of multinozzle electrospray ionization (MnESI) setups: (A) Bar plot showing the average number of identified peptides for each sample loading for 5 μL/min flow rates with and without using a MnESI source. (B) Same as (A) but for protein groups. (C) Same as (A) but for 1.5 μL/min. (D) Same as (B) but for 1.5 μL/min. (E) Violin plots showing the distribution of the relative boost of peptide intensities (based on AUCs of XICs as above) of MnESI compared to the reference setups (optimal peptide loadings of 2 μg for the 5 μL/min and 1 μg for the 1.5 μL/min setups were used). (F) Box plots showing the distributions, medians, and interquartile ranges of chromatographic peak widths at half-maximal signal (FWHM) of identified peptides at optimal loading. (G) Cumulative density plot illustrating the quantitative repeatability (precision) of the capLC setups with and without multinozzle emitters at optimal peptide loading.

### Robustness and Repeatability of the 1.5 μL/min capLC-MS/MS Setup

Given the overall excellent performance of the 1.5 μL/min capLC setup and the fact that this flow rate has been underexplored in the proteomics field, all further experiments were performed using this configuration. To assess its technical robustness and repeatability, we conducted an experiment consisting of 100 consecutive injections, divided into four identical cycles of 25 injections each (Figure 4A), including different sample types and spanning 4.5 days of measurement. Each cycle included 11 replicates of the same HeLa cell line digest, four replicates of the same cerebrospinal fluid (CSF) digest, and 10 replicates of the same human plasma digest. For each sample, 1 μg peptide loading was used, and 300 fmol of a synthetic peptide retention time standard mix (PROCAL)^28^ was spiked into each sample to monitor retention time stability. Blank runs were included between each sample type to evaluate sample carry-over. Analysis of PROCAL peptide retention times revealed peptide-specific CVs of 0.2–1.8% (average 0.7%), demonstrating high chromatographic reproducibility (Figure 4B). Excellent separation robustness, in turn, also resulted in high repeatability of the number of peptide (CV <2%) and protein group (CV <3%) identifications (Figure 4C,D). Identifications achieved for plasma were comparable to state-of-the-art single-shot, unfractionated sample analysis reported in the literature.^29^ For CSF, the number of identified peptides and proteins was almost twice as high as reported in a recent ring trial study.^30^ This may be attributable to the higher loading amount used here (1 μg vs 400 ng), the fact that we pooled CSF from 10 donors, and the possibility that our CSF samples were not free of tissue material that could easily be introduced during lumbar CSF collection. Quantitative precision was also outstanding, with CVs of <20% for 96% of quantified proteins for HeLa and 98% for CSF and plasma samples (Figure 4E). In addition, sample carry-over was very low (0.12% peptide intensity for HeLa, 0.11% for CSF, and 0.48% for plasma; calculated by dividing the total intensities of identified peptides in a blank run by that of the previous sample run), demonstrating that the 1.5 μL/min capLC system is a highly effective setup for analyzing these sample types when using optimal sample loading.

**Figure 4:**
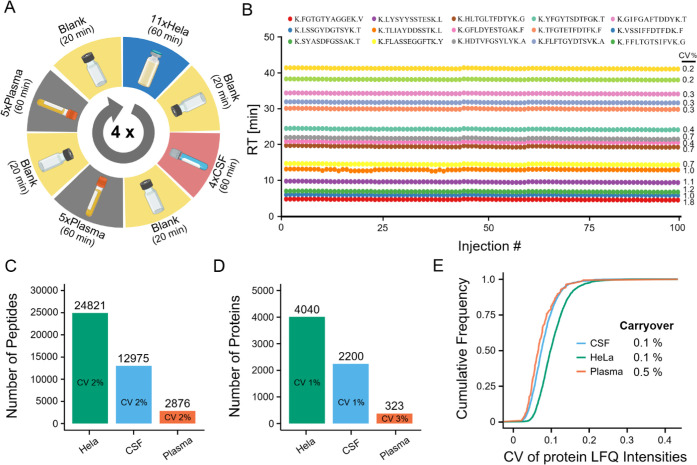
Robustness and repeatability of the 1.5 μL/min capLC setup: (A) Scheme illustrating the design of the robustness and repeatability test. (B) Retention time (RT) plots of 15 PROCAL peptides and their associated average retention time CVs across 100 consecutive sample injections. (C) Bar plot summarizing peptide identification stability across the cycle shown in (A). (D) Same as (C) but for protein groups. (E) Cumulative density plot illustrating the quantitative repeatability (precision) as well as run-to-run average sample carryover of the different sample types.

### Example Applications for capLC-MS/MS in Proteomics

The above shows that the 1.5 μL/min capLC system can be used to analyze samples as diverse as plasma or cell lines. However, for plasma in particular, it can be argued that μLC is the more convenient choice because protein is easily and abundantly available (∼60 μg/μL), and the absolute sample loading required to achieve good protein coverage is far below the capacity of 1 mm i.d. columns, thereby ensuring high-performance separations over long periods of time. At the other end of the spectrum, the proteome analysis of rare or single-cell populations will require nLC due to absolute sensitivity demands. An interesting middle ground is the analysis of subproteomes that can be enriched biochemically, with two illustrative cases exemplified below. In the first application, we purified phosphopeptides from 500 μg of a HeLa cell line digest by immobilized metal affinity chromatography (IMAC; two workflow replicates). Three samples of 500 ng each were analyzed using either capLC or direct injection nLC-MS/MS systems. Interestingly, the capLC setup identified ∼10,000 phosphopeptides, outperforming the nLC setup by 21% (11% at the level of phosphoproteins; Figure 5A,B). This difference is likely due to the five min shorter gradient time available on the direct injection nLC setup compared to the capLC system. As mentioned above, the sharper chromatographic peaks of the capLC setup also contribute to this performance advantage (Figure 5C) as peptide intensities, and Andromeda scores were comparable between the two setups (Figures S6A and 5D).

**Figure 5:**
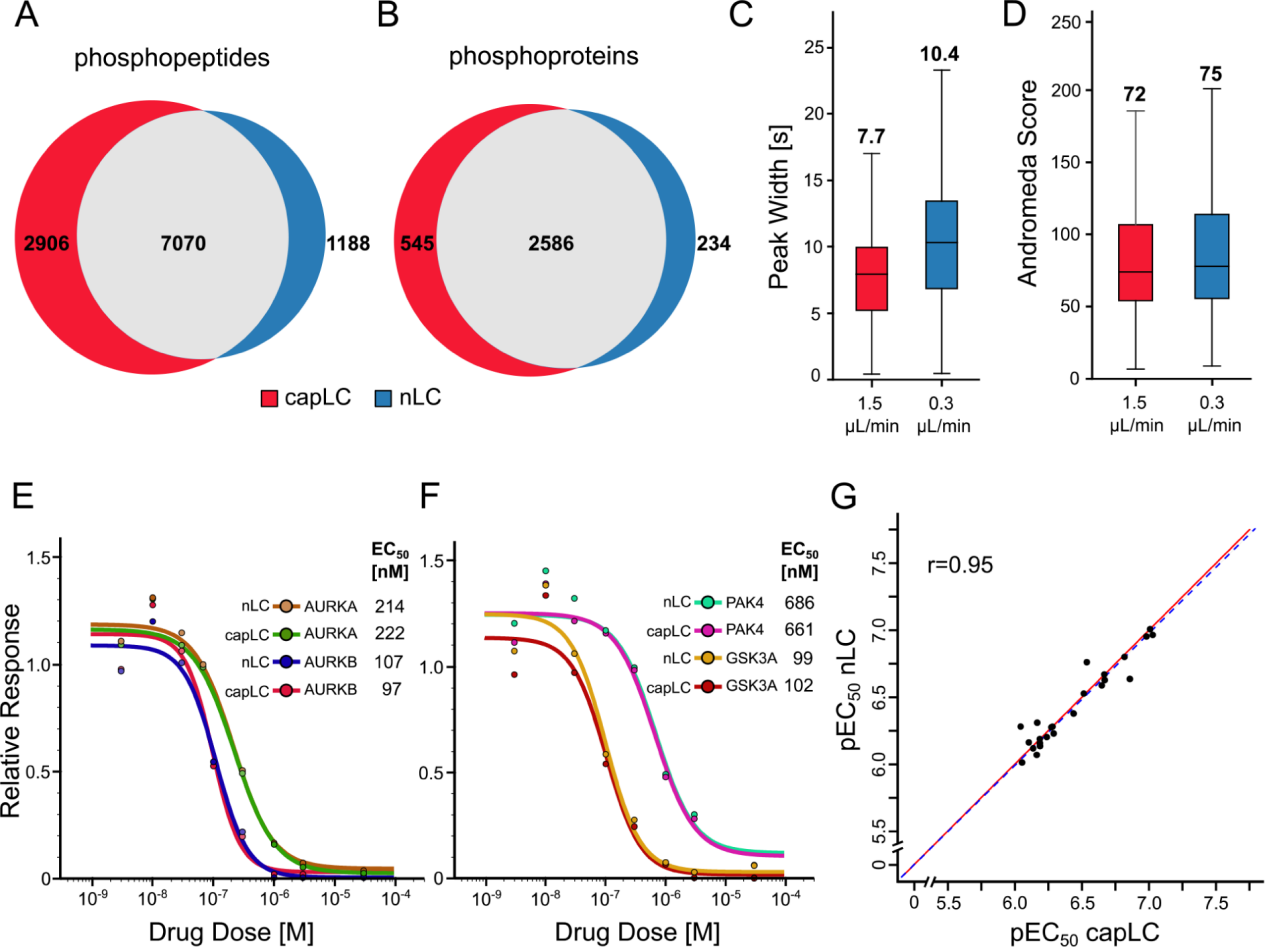
Comparative analysis of subproteomes on the 1.5 μL/min capLC and nLC systems: (A) Venn diagram comparing the number of identified phosphopeptides. (B) Same as (A) but for phosphoproteins. (C) Box plots showing the distributions, medians, and interquartile ranges of chromatographic peak widths at half-maximal signal (FWHM) of identified phosphopeptides. (D) Box plots showing the distributions, medians, and interquartile ranges of MaxQuant Andromeda scores of all phosphopeptides. (E,F) Dose–response curves illustrating the interaction of the kinase inhibitor AT-9283 with a number of target proteins (EC_50_: effective concentration to reduce 50% of protein binding to kinobeads). (G) Scatter plot correlating pEC_50_ values (≥6; equivalent to EC_50_ < 1,000 nM) measured for drug-target interactions obtained by the capLC and nLC setups. *r* = Pearson correlation coefficient, fitted regression line in blue, and *x*-*y* diagonal line in red.

In a second example, we compared the capLC to nLC-MS/MS setups for analyzing drug–protein interactions using the multikinase inhibitor AT-9283 and the kinobeads approach.^31^ The capLC and nLC systems identified 289 and 287 kinases, respectively (Figure S6B). CurveCurator^23^ was used to analyze the dose–response data and determine apparent interaction constants (*K*_d_^app^) for the compound and its target proteins. This analysis resulted in the identification of 58 and 54 targets using capLC- or nLC-MS/MS, respectively (Figure S6C). More importantly, overlaying the dose–response curves obtained by both analytical setups showed nearly identical results for the potent drug targets (e.g., AURKA, AURKB, GSK3A, and PAK4; Figures 5E,F and S7). Consequently, their determined half-maximal effective concentration (EC_50_) and *K*_d_^app^ values were also very similar. This was also more generally true when correlating the pEC_50_ values (−log_10_EC_50_; Pearson correlation coefficient *r* = 0.95; Figure 5G) or p*K*_d_^app^ values (−log_10_ *K*_d_^app^; *r* = 0.88; Figure S6D) obtained by capLC or nLC-MS/MS, respectively, for all commonly identified targets.

## Conclusions

In this study, we conducted a side-by-side performance evaluation of μLC, capLC, and nLC-MS/MS systems (0.3 μL/min to 50 μL/min) for use in proteomics. The data confirmed many previously reported results^9,18^ but also addressed a gap in the literature by systematically evaluating all flow rates and column dimensions useful for standard proteomic applications, largely eliminating the influence of the particular sample preparation, mass spectrometer, or data analysis software employed. A limitation of the current study is that it did not evaluate throughput scenarios higher than 24 SPD. However, we consciously chose to perform the evaluation using a 60 min turnaround time because the effective gradient times of between 45 and 54 min are long enough to ensure that all the data are of high qualitative and, more importantly, quantitative quality. We also consciously chose a DDA over a DIA approach and refrained from employing performance-enhancing data processing tools such as match-between-runs^32^ or AI-based identification rescoring approaches such as PROSIT^33^ across different flow rates or sample loadings for the sake of being conservative and avoiding additional parameters unrelated to the chromatographic part of the overall analytical proteome analysis workflow. Still, the authors anticipate that the observations and learnings made for the 24 SPD data will translate to shorter gradients, as long as the employed mass spectrometer can keep up with the sharper LC peaks and higher number of coeluting peptides at shorter gradients.^34–36^ The commercial availability of HPLC systems that can accommodate a wide range of flow rates facilitates the choice of the right column and gradient for the right application, especially for laboratories that cannot reserve dedicated LC-MS/MS systems for particular applications. We also anticipate that this study will provide useful practical guidance for scientists working in the field on which setup to choose for a given application.

## Supplementary Material





## Data Availability

The mass spectrometry proteomics data and MaxQuant search results have been deposited with the ProteomeXchange Consortium via the PRIDE^25^ partner repository. Project accession: PXD062536; Token: 4 °Cpdr2cNZ99. The source data underlying Figures 2 A,B; 3 A–D; 4 B–E; 5G; and S6D are provided as a supplementary source data file.

## Notes

The following files are available: Supplementary Figures: number of average MS/MS scans collected in capLC vs nLC-MS/MS (Figure S1); representative base peak chromatograms for each LC setup (Figure S2); relative boost of peptide intensity distributions for all LC setups at different peptide loadings compared to the reference μLC setup and box plots showcasing the distributions of MaxQuant Andromeda scores of all setups at different peptide loadings (Figure S3); estimated peak capacities of all setups (Figure S4); comparison of peptide and protein identifications in replicate measurements of each LC-MS setup (Figure S5); relative boost of peptide intensities of capLC vs nLC setups, Venn diagrams comparing capLC vs nLC in terms of the number of detected kinases and also known target proteins of kinase inhibitor AT-9283 in kinobeads pulldown experiments, scatter plot correlating p*K*_d_^app^ calculated from dose–response curves obtained by the capLC and nLC setups (Figure S6); dose–response curves and EC_50_ values for all targets of the kinase inhibitor AT-9283 identified by capLC, nLC, or both setups (Figure S7) (PDF)
Supplementary Source Data: it contains source data used for creating Figures 2A,B; 3 A,B,C,D; 4B,C,D,E; 5G; and Figure S6 (XLSX)

## Abbreviations

ACN: Acetonitrile
AUC: Area under the curve
CAA: Chloroacetamide
capLC: Capillary liquid chromatography
CSF: Cerebrospinal fluid
CV: Coefficient of variation
DDA: Data-dependent Acquisition
DIA: Data-independent Acquisition
DMSO: Dimethyl sulfoxide
DTT: Dithiothreitol
EC_50_: Effective concentration to reduce 50% of protein binding to kinobeads
FBS: Fetal bovine serum
fmol: Femtomole
FDR: False discovery rate
FWHM: Full width at half maximum
HeLa: Human cervical carcinoma cell line
HLB: Hydrophilic–lipophilic balance
HPLC: High-performance liquid chromatography
i.d: Internal diameter
*K*_d_^app^: Apparent dissociation constant
LC: Liquid chromatography
LC-MS/MS: Liquid chromatography-tandem mass spectrometry
μg: Microgram
min: Minute
μL: Microliter
μLC: Microflow liquid chromatography
mm: Milimeter
mM: Milimolar
MS: Mass spectrometry
Ng: Nanogram
nL: Nanoliter
nLC: Nanoflow liquid chromatography
nM: Nanomolar
PSM: Peptide-spectrum match
RT: Retention time
SPD: Samples per day
SWATH-MS: Sequential window acquisition of all theoretical mass spectra
TFA: Trifluoroacetic acid
Å: Angstrom
